# Attitude towards Mental Illness among Primary Healthcare Providers: A Community-Based Study in Rural China

**DOI:** 10.1155/2018/8715272

**Published:** 2018-09-30

**Authors:** Zhenyu Ma, Hui Huang, Guanghui Nie, Vincent M. B. Silenzio, Bo Wei

**Affiliations:** ^1^School of Public Health, Guangxi Medical University, Nanning, China; ^2^Injury Control Research Center for Suicide Prevention, Department of Psychiatry, University of Rochester Medical Center, Rochester, USA

## Abstract

**Objective:**

There are no studies that have explored attitudes towards mental illness that are held by rural primary healthcare (PHC) providers. The aim of this study was to conduct evidential and comparative research about attitudes towards mental illness among primary healthcare providers from different mental health service models in China rural communities.

**Methods:**

A self-administered questionnaire was conducted with a total of 361 rural primary healthcare providers engaged in mental health service delivery.

**Results:**

Total attitude score mark of rural primary healthcare providers shows that most PHC providers still held pessimistic and negative attitude towards mental illness patients. 71.3% of respondents agreed that “the mental patients often impulsively perform destruction of property”; 72.9% agreed that “mental patients are burdens to the families and society.” There are also positive correlations between attitudes and abilities of primary healthcare providers to mental illness.

**Conclusion:**

This study provides baseline evidence that primary healthcare providers in rural China hold negative attitudes towards mental illness. It is critical to improve negative attitudes and understanding about the importance of the management of severe mental illness among rural primary healthcare workers in mental health services. We should take comprehensive methods to enrich primary healthcare providers' professional knowledge about mental illness and eliminate discrimination and inappropriate perception against the mental illness.

## 1. Introduction

Integrating mental health services into primary care World Health Organization (WHO) report showed that integrating mental health services into primary care generates good health outcomes at reasonable costs. It is the most viable way of closing the treatment gap and ensuring that people get the mental healthcare they need [1]. That means mental health services should be delivered by the primary healthcare (PHC) providers rather than psychiatrists. Mental health in China is a great public health concern given the large number of patients and huge social and economic implications. In 2009, the new healthcare reform integrated severe mental illnesses into the national primary public health services and stipulated that PHC providers should manage patients with severe mental illness [2]. Essential services at this level include early identification of mental disorders, management of stable psychiatric patients, referral to other levels where required, and promotional and prevention activities. Now the PHC provider is the largest group of mental health service workforce, so their attitudes towards mental illness may affect the quality of mental health services [3].

Guangxi is a less developed area in China with a lack of mental health professionals, especially in rural communities. Due to the shortage and urban-centered unequal distribution of mental health professionals, the improvement and development of community mental healthcare has to rely on the primary healthcare providers, also known as “village doctors” who are usually from the same community and are familiar with the community members in the area [4]. Mental healthcare provision should be switched from traditional psychiatric hospitals-centered pattern to hospital-community based service pattern.

Most previous studies regarding the attitude towards mental illness were conducted in the general population [5, 6] and medical students [7, 8]. However, little is known in our own as well as in other cultures about the attitudes PHC providers have about mental illness and those affected. Thus, in this study, firstly we assessed attitudes towards mental illness in a sample of Chinese rural PHC providers. Secondly, we compared the outcomes among two different mental health service models in rural Guangxi: one is hospital-community integrated service model and the other is the psychiatric hospital-centric model. To the best of our knowledge, this is the first study to examine the attitudes towards mental illness among primary healthcare providers who provide the majority of first-line care.

## 2. Methods

### 2.1. Sample Area Selection and Participants

We selected participants from the two different mental health service model areas in rural Guangxi. The sample area is Liujiang County, with a new hospital-community integrated service model; this program covers 500000 general population in 11 towns. The National Continuing Management and Intervention Program for Psychoses (686 Program) has been developed in Liujiang since 2006. However, Liucheng County is the control area in this study, and its socioeconomic development level and capacity of rural health services are comparable with Liujiang County, and it still follows the traditional hospital-centric model of community mental health services [4].

We randomly selected 3 towns from Liujiang County according to the socioeconomic status of high, medium, and low. Also, we arranged 3 towns in Liucheng County to compare with Liujiang County in accordance with the socioeconomic situation. There were a total of 6 towns from these 2 sample counties. The participants were all of the 316 primary healthcare providers who deliver mental healthcare in the 6 towns.

### 2.2. Research Content and Tools

The interview schedule was divided into two parts. Part I included the sociodemographic data: age, sex, education background, job title, income, and willingness to work also collected. Part II included Mental Illness Attitude Questionnaire developed by Beijing Hui Long Guan Hospital [9], which consisted of six subscales: (1) causes of mental illness, (2) violence and aggression of mental illness patients, (3) hope for psychiatric treatments, (4) social value of mental illness patients, (5) problems of avoidance of mental illness patients, and (6) restrictions on mental illness patients. Each subscale has 5 items. The Mental Illness Attitude Questionnaire has 30 items in total, using a 4-point scale (1= strongly disagree, 4=strongly agree) to measure attitudes towards causes, treatment, and its impact on individuals and society. Add the scores of all the 5 items in each subscale to get a total score ranging from 5 to 20 and then put it into the form of a percentage to the maximum subscale score. Use the numerator of this percentage (25-100%) as the value of each subscale for interviewee to make statistical analysis. The score of each subscale ranges from 25 to 100. Higher score indicates more agreement with the attitude represented in the subscales. The questionnaire had been tested with high content consistency and test-retest reliability [10].

### 2.3. Ethics Statement

In order to eliminate possible bias and encourage participants to report their true attitude, the survey was designed as anonymous. The potential risks and benefits of the survey were described by the interviewers. Interviewers were graduate students at the School of Public Health of Guangxi Medical University and attended training courses on research methods and rural mental health services research. The training also included a session on the ethical aspects of human subject research. To compensate for their time, each participant was given a cash amount of RMB 50 (US$8). The study was approved by the Ethics Committee of the Guangxi Medical University.

### 2.4. Statistical Analyses

Statistical analysis was conducted using SPSS 19.0.* t-*test, analysis of variance, *χ*2 test, Fisher exact test, and Wilcoxon test were performed to examine the differences between two groups.* P*<0.05 was considered with statistically significance.

## 3. Results

### 3.1. Sample Characteristics

A total of 316 PHC providers in the six towns agreed to participate in the study, and 291 of them completed all interviews. The response rate was 92.1%. 158 completers (54.3%) were from Liucheng County and 133 (45.7%) from Liujiang County. The sex distribution in the completers was 170 males (58.4%) and 121 females (41.6%). The mean age was 44.6 (SD = 1.8), and the mean number of working years was 21 (detailed in Table 1).

### 3.2. Attitude towards Mental Illness among PHC Providers in Rural Community

The results showed that most PHC providers still held pessimistic and negative attitude towards mental illness patients. The 4, 5, and 6 subscales described the negative attitude: mental illness patients cannot contribute to society; people should avoid mental illness patients and their family members; mental illness patients' social activities should be limited, with the score were between 63.9±6.9 and 54.9±8.4. It can be inferred that PHC providers held different levels of pessimistic and negative attitude. Meanwhile, from the other three subscale scores, the rural community PHC providers also had some misunderstandings to the awareness of mental illness.* t-*test was performed to examine the attitude subscale scores and the difference was not statistically significant (P>0.05) (detailed in Table 2).

*χ* ^*2*^ test was also performed on each of the 30 psychiatric statements in the Mental Illness Attitude Questionnaire (see details in Appendix). Between two counties, there were only three items that showed statistically significant differences, which were item 10 “the violence acts of mental illness patients were as much as the others” (*P* = 0.011), item 12 “mental illness can be treated” (*P *<0.001), and item 16 “mental illness patients are able to contribute to society” (*P* = 0.037).

We also found high percentage of agreement in some negative attitude statements: 71.3% of PHC providers agreed that “mental illness patients often impulsively perform destruction of property,” 72.9% agreed that “mental illness patients are burdens to the families and society,” etc. It is worth mentioning that over 92% of PHC providers were in favor of “drugs can control the symptoms of mental illness” and “rehabilitation of mental illness is very effective,” which indicated that PHC providers have recognized the importance of psychotropic drugs to control severe mental illness.

### 3.3. Attitudes and Abilities of PHC Providers to Mental Illness

We examined correlations between attitudes and abilities of PHC providers to mental illness (see Table 3). The more agreement of “mental illness patients cannot contribute to society” and “people should avoid mental illness patients and their family members,” the lower mental health service capacity the PHC providers had.

## 4. Discussion

The findings suggest that the attitudes of PHC providers were negative towards the mentally ill, no matter whether in hospital-community integrated service model or psychiatric hospital-centric model. Nowadays, in the era of rapid development of technology and civilization, there has been some improvement in tolerance and attitudes. However, there are still many people who have pessimistic and negative attitude towards mental illness, including PHC providers themselves. Some think that mental disorders cannot be treated, but we know that effective treatments exist and can be successfully delivered in primary care. Some believe that people with mental disorders are violent or unstable and therefore should be locked away, while in fact the vast majority of affected individuals are nonviolent and capable of living productively within their communities. Some evidence shows that different educational level [11], healthcare systems, and cultural differences [12] seem to have an impact on differences in attitudes towards people with mental illness.

Our results found high agreement in subscale “violence and aggression of mental illness patients.” 71.3% -83.7% of PHC providers agreed that “mental illness patients often appear impulse, destroy behaviors,” “mental illness patients often lose their temper with no reason,” and “mental illness patients often appear unexpected impulsive behaviors.” The most common response to mental illness is fear of dangerousness. People hold negative attitudes towards mental illness, particularly, a perception that someone with mental illness is dangerous [13]. For example, the typical statements of PHC providers said in our previous qualitative study were “Frankly speaking, I am afraid of mental disorders' attack. Especially the manic patients. Even though it is dangerous, it is still my duty to contact with them” and “I do not like to contact with mental disorders. They are abnormal and maybe dangerous” [4]. In addition, this study also found that more PHC providers in Liujiang County thought that “the violence acts of mental illness patients were as much as the others,” “mental illness can be treated,” and “mental illness patients are able to contribute to society.” The different attitudes maybe due to the administration of mental illness in Liujiang County were earlier and longer, which have more training and implementary programs. This is also consistent with the findings of another study in China [14], which reported that, after providing free medication, the majority of patients can get control of the illness, some of which can reintegrate or do some simple productive labor. In Liujiang County, some patients could go back to work and farm after free medication. Therefore, PHC providers in Liujiang County have more recognition of the statement that “mental illness patients are able to contribute to society.”

The attitudes towards the mental illness patients will greatly affect the practice of PHC providers. Attitude, as a way to assess the psychological constitution, is relatively long-lasting and stable, and it can predict impact behavior to some extent [15]. Positive and correct attitude can make providers care for patients and provide active treatment, health education, and rehabilitation guidance. Negative attitudes towards mental illness are known to be associated with poorer patient functioning and overall client outcomes. Although PHC providers deliver mental health services for mental disorders, their negative attitudes towards mental illness may affect the ways of mental health services, such as passive work and reluctance; this also may affect patients' self-evaluation and treatment compliance, thus reducing the ultimate effects of the treatment PHC providers provide.

This study presents baseline data from the rural Chinese context that adds to the evidence on PHC providers' attitude to mental illness. Considering the results of the present study, it seems that comprehensive strategies are necessary, to enrich primary healthcare providers' professional knowledge about mental illness and eliminate discrimination and inappropriate perception against mental illness, no matter whether in hospital-community integrated service model or psychiatric hospital-centric model. We recommend that, first of all, the professional ideological education of the PHC providers should be strengthened, so that they will consciously serve, care, and respect the patients. Second of all, professional continuing education and training should be actively developed, in order to improve the level of mental health awareness for PHC providers [16]. This is critical to improve PHC providers understanding about the importance of the management of severe mental illness and enrich PHC providers' professional knowledge about mental illness, so that they will have confidence and hope in their future development. There is also a need to weaken and eliminate discrimination and inappropriate perception against mentally ill patients and mental illness by means of community education. To enable understanding and supportive social environment for the mentally ill patients will contribute to the establishment of a good attitude for PHC providers and will be more conducive to the development of community mental health services.

## Figures and Tables

**Table 1:** Demographic characteristics of PHC providers in two sample counties.

Demographic Variable	Freq. (%)		*χ* ^*2*^ */t*	*P*
	Liu Cheng (N=158)	Liu Jiang (N=133)		
Sex				
Male	82(51.9%)	88(66.2%)	6.051	0.014
Female	76(48.1%)	45(33.8%)		
Age	45.06±14.18	44.08±11.47	0.657	0.512
Education				
Junior high school or below	43(27.2%)	19(14.3%)	14.736	0.002
Senior high school	73(46.2%)	90(67.7%)		
Junior college	32(20.3%)	19(14.3%)		
College or above	10(6.3%)	4(3.0%)		
Job Title				
No title	113(71.5%)	100(75.2%)	0.512	0.774
Primary	36(22.8%)	26(19.5%)		
Intermediate or above	9(5.7%)	7(5.3%)		
Years of Work				
Less than 10 years	40(25.3%)	32(24.1%)	3.928	0.269
11~20 years	41(25.9%)	46(34.6%)		
21~30 years	30(19.0%)	26(19.5%)		
More than 30 years	44(27.8%)	26(19.5%)		
Major				
Clinical Medicine	82(51.9%)	79(59.4%)	6.511	0.089
Traditional Chinese medicine	14(8.9%)	7(5.3%)		
Preventive medicine	21(13.3%)	26(19.5%)		
Others	36(22.8%)	19(14.3%)		
Monthly Income				
Less than 1000RMB	97(61.4%)	46(34.6%)	42.221	<0.001
1001~2000 RMB	54(34.2%)	49(36.8%)		
2001~3000 RMB	3(1.9%)	25(18.8%)		
More than 3000 RMB	2(1.3%)	13(9.8%)		
Willing to provide mental health services				
Yes	126(79.7%)	125(94.0%)	10.977	0.001
No	28(17.7%)	7(5.3%)		

**Table 2:** The scores of PHC providers' attitude towards mental illness.

Attitude subscales	Liucheng (n=158) $\overset{-}{x}\pm s$	Liujiang (n=133) $\overset{-}{x}\pm s$	Total (n=291) $\overset{-}{x}\pm s$
(1) Mental illness is caused by biological factors	67.9±8.8	67.4±8.7	67.6±8.7
(2) Mental illness patients' behaviors are violent and aggressive	69.4±9.2	69.9±8.1	69.6±8.7
(3) Mental illness is incurable	54.4±7.7	55.2±7.0	54.7±7.4
(4) Mental illness patients cannot contribute to society	64.6±7.0	63.2±6.8	63.9±6.9
(5) People should avoid Mental illness patients and their family members	54.5±8.2	55.3±8.6	54.9±8.4
(6) Mental illness patients' social activities should be limited	60.5±10.3	58.4±8.7	59.6±9.6

Note: means and standard deviations in the table are the percentages of the answered scores divided by the total score of subscales (the total score of each subscale is 20). The higher the percentage of means, the more agreement with the statements of the subscale.

**Table 3:** Correlation analysis of attitudes and abilities of the primary healthcare providers to mental illness.

Spearman correlation analysis	Recognition ability	Communication ability	Execution ability	Learning ability	Total score
(1) Mental illness is caused by biological factors	-0.086	-0.001	0.058	0.084	-0.011
(2) Mental illness patients' behaviors are violent and aggressive	0.065	-0.008	0.006	0.010	0.077
(3) Mental illness is incurable	-0.026	-0.071	0.006	0.045	-0.025
(4) Mental illness patients cannot contribute to society	0.064	-0.144^*∗*^	-0.161^*∗∗*^	-0.201^*∗∗*^	-0.118^*∗*^
(5) People should avoid Mental illness patients and their family members	0.040	-0.094	-0.050	-0.057	-0.119^*∗*^
(6) Mental illness patients' social activities should be limited	0.077	-0.161^*∗∗*^	-0.147^*∗*^	-0.129^*∗*^	-0.110

^*∗∗*^ *P*<0.001,^*∗*^ *P*<0.05

**Table 4:** Mental Illness Attitude Questionnaire (developed by Beijing Hui Long Guan Hospital).

Items
**Causes of mental illness**
Mental illness is caused by family problems
Mental illness is caused by patients' own mistakes
Mental illness is caused by the brain diseases
Mental illness is caused by social interpersonal tension
Mental illness is caused by the problems of myth
**Violence and aggression of Mental illness patients**
Mental illness patients often appear impulse, destroy behaviors
Mental illness patients often threaten or harm the people around
Mental illness patients often lose their temper with no reason
Mental illness patients often appear unexpected impulsive behaviors
Violence of Mental illness patients is as much as that of others
**Hope for psychiatric treatments**
Mental illness can be cured
Mental illness can be treated
Symptoms of Mental illness patients can be controlled by drugs
Treatments of psychiatric rehabilitation are very effective
Difficult to treat mental illness
**Social value of Mental illness patients**
Mental illness patients can contribute to society
Mental illness patients often disturb public order
Mental illness patients are burdens to families and society
Mental illness patients violate social and moral rules as much as other people do
Mental illness patients are useless to society
**Problems of avoidance of Mental illness patients**
Those who regularly contact with Mental illness patients will suffer from mental problems
Mental illness patients are not able to deal with the world
Best not to marry the relatives of Mental illness patients
Dealing with Mental illness patients make people being humiliated or disgraced
Regular contacts with Mental illness patients do not have any harm
**Restrictions on Mental illness patients**
Those who have history of mental illness should not get married
Those who have history of mental illness should not give birth
Discharged mental illness patients should be allowed to return to society
Those who have history of mental illness should be allowed to access to universities
Those who have history of mental illness should be allowed to access to work

## Data Availability

The data used to support the findings of this study are available from the corresponding author upon request.

## Appendix

See Table 4.
